# A hard permanent magnet through molecular design

**DOI:** 10.1038/s42004-021-00509-y

**Published:** 2021-05-19

**Authors:** Ryan A. Murphy, Jeffrey R. Long, T. David Harris

**Affiliations:** 1 Department of Chemistry, University of California, Berkeley, CA USA; 2 Department of Chemical and Biomolecular Engineering, University of California, Berkeley, CA USA; 3 Materials Science Division, Lawrence Berkeley National Laboratory, Berkeley, CA USA

**Keywords:** Magnetic materials, Metal-organic frameworks

## Abstract

Permanent magnets constructed from metal ions and organic linkers using molecular design principles could bring transformative advances in areas such as energy conversion, transportation, and information storage. This comment highlights the recent discovery of a metal–organic magnet ordering at 242 °C, and discusses future research directions and possible applications involving such materials.

Permanent magnets—materials that can retain their magnetization in the absence of an applied magnetic field—enable myriad technologies underpinning modern society^1^. They serve as critical components of the speakers in nearly all electronic devices, of the electric motors in household appliances, and of the information-storage media in computers^2^. Moreover, magnets play a key role in our evolving energy landscape, forming the central operative elements of inductive generators in wind turbines and regenerative brakes in electric cars^3^. Despite this importance to numerous established and emerging technologies, progress in developing new permanent magnets with improved properties has stagnated. For instance, the intermetallic compound Nd_2_Fe_14_B, discovered in 1984, remains the strongest commercial magnet and is used in many of the aforementioned applications^4,5^. This dearth of progress in discovering new magnets stems in part from the empirical approach historically used to synthesize inorganic materials, which constrains the design of materials with targeted structures and properties.

The limitations associated with conventional solid-state magnets have prompted researchers to explore an approach in which molecular building units, namely metal ions and inorganic or organic linkers, are combined in solution to produce an extended coordination solid. In principle, judicious selection of metal ion and linker can give materials with programmed structures and magnetic properties. Moreover, such magnets derived from discrete molecular precursors are amenable to solution processability, a property that is exceedingly difficult to realize for solid-state magnets. Much of the foundational research in the development of molecule-based magnets focused on extended solids of metal ions or complexes and radical-based organonitrile^6^ or nitronyl nitroxide linkers^7^. The use of radicals was envisaged to provide the strong magnetic coupling between spin centers needed for high-temperature magnetic order. Indeed, the amorphous material V(TCNE)_~2_·~0.5CH_2_Cl_2_ (TCNE = tetracyanoethylene) was shown to exhibit magnetic order up to its thermal decomposition at 77 °C, thereby representing the first room-temperature molecule-based magnet^8^. Nevertheless, the instability and weak magnetic performance of this material have prevented its translation to the energy industry. Metal-substituted analogs of the centuries-old coordination compound Prussian blue, Fe^III^_4_[Fe^II^(CN)_6_]_3_·*z*H_2_O, have also been pursued as high-temperature permanent magnets^9^. Indeed, the compound KV^II^[Cr^III^(CN)_6_]·2H_2_O magnetically orders at 103 °C^10^, yet antiferromagnetic coupling between isotropic and isoelectronic metal ions results in leads to only miniscule magnetic hysteresis at room temperature.

As the discovery of new routes for synthesizing molecule-based magnets with high ordering temperatures waned, metal–organic frameworks were rapidly emerging. These materials feature open structures comprising metal ions or clusters that are connected together by anionic organic linkers via strong bonding interactions (Fig. 1)^11^. The expansive chemical space associated with framework design is unparalleled in extended solids, and it permits the design of isoreticular metal- and linker-substituted series. In conjunction with this remarkable degree of synthetic control, the lightweight, highly porous structures of frameworks enable their use in a wide range of applications, from gas storage and separations to catalysis^12^. Moreover, these materials often accommodate post-synthetic modification, such as reductive insertions analogous to the soft chemistry developed for layered solid-state compounds^13^, and their kinetically controlled structures can further allow exchange of metal and linker components^14^. For these reasons, metal–organic frameworks are also versatile materials for the directed assembly of designer, high-temperature magnets^15^.

**Fig. 1:**
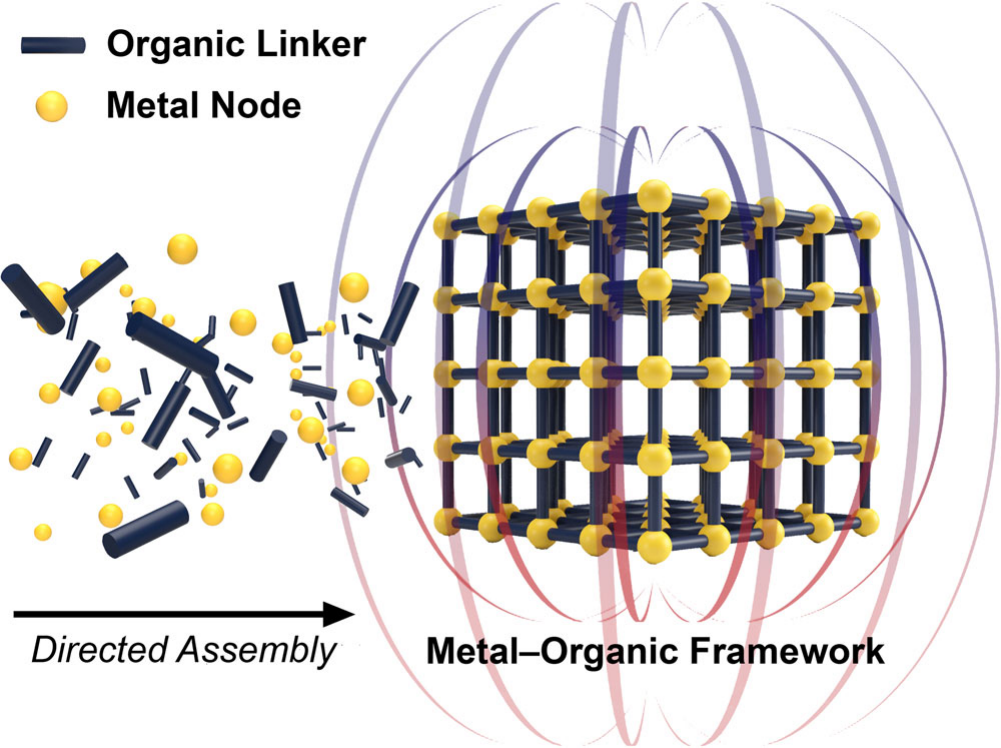
Control of chemical structure and function. The synthetic versatility and predictability associated with metal–organic frameworks can be leveraged to design new permanent magnets.

Recently, Oyarzabal, Clérac, and coworkers reported a remarkable ordering temperature of 242 °C for the crystalline framework material Cr(pz)_2_·0.7LiCl (pz = pyrazine), surpassing V(TCNE)_~2_·~0.5CH_2_Cl_2_ and representing a new record among all molecule-based magnets^16^. This compound was synthesized via chemical reduction of the parent framework *trans*-CrCl_2_(pz)_2_ (Fig. 2), which was obtained through the reaction of CrCl_2_ with pyrazine, conceptionally similar to formation of the molecular complex *trans*-CrCl_2_(py)_4_ (py = pyridine)^17^, and features chloro-terminated Cr^III^ ions bridged by an equal number of formally neutral and radical anionic pz linkers to form 2D sheets^18^. An array of physical methods was deployed on Cr(pz)_2_·0.7LiCl to unambiguously determine that reduction in the Cr(pz)_2_·0.7LiCl product occurs at both Cr and pz, giving a material containing 2D sheets of square planar *S* = 2 Cr^II^ centers linked by pz radical anions. The reduction is accompanied by the loss of 1.3 equivalents of LiCl, giving 2D sheets of Cr(pz)_2_ intercalated with 0.7 equivalents of LiCl. Strong magnetic coupling between spins in energetically similar orbitals of Cr^II^ and pz engenders the unprecedented ordering temperature, and magnetic anisotropy gives rise to magnetic hysteresis with large coercivities, including 0.75 T at 27 °C (Fig. 3). This value constitutes a new record among molecule-based magnets, surpassing that of 60 Oe (0.006 T) for V(TCNE)_~2_·~0.5CH_2_Cl_2_ by over two orders of magnitude. The coercivity—the strength of magnetic field needed to demagnetize a material—in part governs the energy that can be stored or converted by a permanent magnet, and is therefore a key metric to quantify the strength, or “hardness”, of a permanent magnet^1^. Importantly, while the square planar Cr^II^ ion does not formally feature first-order orbital angular momentum, transfer of spin density onto the pz linker may lead to a spin–orbit-coupled magnetic moment of Cr, thereby imparting angular momentum^18^. Such a scenario is consistent with the experimentally observed low magnetization values at 7 T, and may be the primary source of the large coercivity (Fig. 3c).

**Fig. 2:**
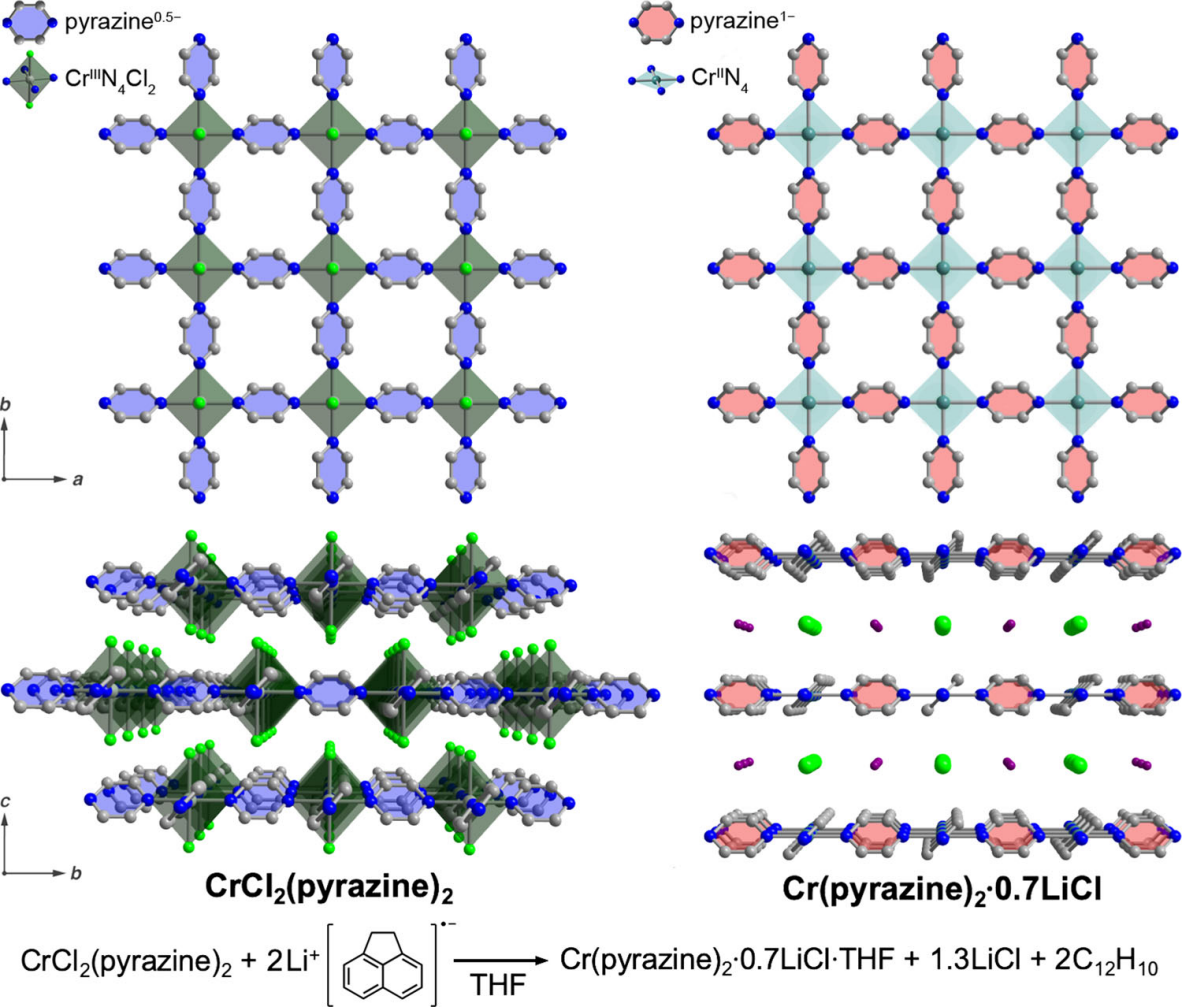
Reductive insertion chemistry in 2D chromium–pyrazine frameworks. Chemical reaction for the reduction of CrCl_2_(pz)_2_ to the high-temperature magnet Cr(pz)_2_, and corresponding crystal structures obtained from X-ray diffraction. Li and Cl atoms are disordered and are modeled with site occupancies of 0.70 and 0.35, respectively.

**Fig. 3:**
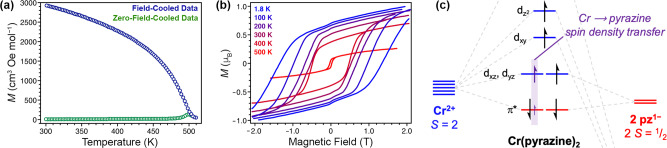
High-temperature, hard magnetism in Cr(pz)_2_·0.7LiCl. **a** A bifurcation in the field-cooled/zero-field-cooled magnetization (*M*) data below 515 K indicates magnetic order. **b** Magnetic hysteresis shows coercivities up to 1.35 T at 1.85 K. **c** Strong magnetic coupling and anisotropy arise from energy matching of molecular fragments. Figures (**a**) and (**b**) were created from the data in ref. ^16^.

Relative to commercial magnets, the coercivity of 0.75 T obtained for Cr(pz)_2_·0.7LiCl at 27 °C compares reasonably well to the room-temperature values of 1.9 T and 4.4 T for Nd_2_Fe_14_B and SmCo_5_, respectively, and is higher than the rare-earth-free hard ferrites (0.40 T), AlNiCo_5_ (0.080 T) and AlNiCo_8_ (0.20 T)^16^. These comparisons are particularly promising considering the lack of formal first-order angular momentum in Cr(pz)_2_·0.7 LiCl. Nevertheless, the corresponding remanence, which quantifies the magnetization of a material remaining upon removal of an applied field and is thus another important parameter in determining magnet performance, of 0.52 μ_B_ per formula unit for Cr(pz)_2_·0.7LiCl, is dwarfed by the analogous values for commercial magnets, such as 32.5 and 8 μ_B_ for Nd_2_Fe_14_B and SmCo_5_, respectively^19^. While this disparity illustrates the need for further advances, the discovery of high-temperature, strong magnetism in a molecule-based material represents a monumental advance, and it underscores the merit of pursuing metal–organic magnets with design features such as covalent bonding between paramagnetic metals and organic linkers.

## Outlook

The modular nature of Cr(pz)_2_·0.7LiCl offers the possibility of making chemical adjustments to target specific structures and properties. Related frameworks with different metal ions, reduced aromatic linkers, terminal axial ligands, or pillaring axial ligands to link the 2D sheets along the c axis, may all be accessible through direct synthesis or post-synthetic substitution chemistry. In particular, the incorporation of metal ions with larger magnetic anisotropy could give rise to a new generation of ultrahard magnets. For instance, the spin–orbit coupling associated with lanthanide and low-coordinate transition metal ions imbues them with immense magnetic anisotropy and large magnetic moments, which are directly correlated to coercivity and remanence, respectively. Indeed, over the past two decades, research in the field of single-molecule magnetism has uncovered how to precisely manipulate the ligand field of certain lanthanide ions to give complexes with record molecular coercivities^20^, yet such molecules have not been chemically linked to give the strong coupling necessary for long-range magnetic order.

The discovery of room-temperature hard magnetism in a low-density material could promote several technological advances. For instance, lightweight hard magnets could replace dense rare-earth magnets to improve energy efficiency in automotive and power conversion applications^3^. In addition, these magnets could find use in sensing media or even in magnetic gas separations, such as the separation of paramagnetic O_2_ (*S* = 1) from diamagnetic N_2_ (*S* = 0)^21^. Along these lines, upon reduction of CrCl_2_(pz)_2_ to Cr(pz)_2_·0.7LiCl, the 2D sheets shift from a staggered to an eclipsed conformation along the c axis, giving rise to tetragonal channels (Fig. 2). Moreover, heating a crystalline sample of THF-solvated Cr(pz)_2_·0.7LiCl led to partial desolvation of THF with no loss of crystallinity and an increase in coercivity. These observations suggest that the partially or fully desolvated sample may show permanent porosity, wherein the framework exhibits measurable surface area upon desolvation, and it highlights the possibility of connecting the 2D sheets at fixed distances with pillaring ligands.

The presence of stacked, neutral 2D sheets in Cr(pz)_2_·0.7LiCl suggests that exfoliation may be readily accessible. Simple chemical methods, such as soaking Cr(pz)_2_·0.7LiCl in a solution of a Li^+^-sequestering crown ether or cryptand ligand, may effect deintercalation of the LiCl sheets. Alternatively, chemical reduction of CrCl_2_(pz)_2_ in the presence of a halide-abstracting agent may provide a route to directly access Cr(pz)_2_. Such exfoliation could be used to cast thin layers of the material onto substrates for numerous studies, potentially allowing for magnetism to be studied as a function of thickness and twist angle down to the monolayer limit using magneto-optical spectroscopy^22^. In general, the development of solution-processable layered magnets synthesized from the bottom up would represent an enormous scientific advancement, with direct utility in nanoscale information storage and other spintronics applications^23^.

Since the discovery of electron delocalization in the archetypal Creutz–Taube ion^24^, [(NH_3_)_5_Ru(pz)Ru[(NH_3_)_5_]^5+^, the capacity of a bridging pyrazine ligand to mediate strong electronic coupling and delocalization within mixed-valence metal complexes has been widely studied^25^. In tetragonal MX_2_(pz)_2_ frameworks, certain mixed-valence combinations of metal and linker could lead to both a high electronic conductivity and a high magnetic ordering temperature. Further, electron delocalization in a mixed-valence species with more than one unpaired spin can enforce exceptionally strong ferromagnetic alignment of spins via a double-exchange mechanism, analogous to conventional permanent magnets^26^. Indeed, such itinerant ferromagnetism was recently observed to promote magnetic order up to –48 °C in a chromium(II/III) triazolate framework^27^. The parent material CrCl_2_(pz)_2_ exhibits a high room-temperature conductivity of 32 mS cm^–1^, which is attributed to electron delocalization stemming from linker-based mixed valency. Subsequent reduction to Cr(pz)_2_·0.7LiCl is associated with an approximately hundred-million-fold decrease in conductivity owing to the univalent metal and linker constituents in the latter, suggesting that this and related materials may serve as redox- or light-actuated magnetic semiconductors.
